# 

**Published:** 1909-04-03

**Authors:** 


### BOOKS RECEIVED.

Longmans, Green and Co.

"The Break-up of the Poor Law." Part I. Edited by Sidney and Beatrice Webb.

John Murray.

"The Frontiersman's Pocket-book." Compiled and edited by Roger Pocock.

"The Interpretation of Radium." By Frederic Soddy, M.A.

James Maclehose and Sons, Glasgow.

"Manual of Diseases of the Ear." By Thomas Barr, M.D., and J. Stoddart Barr, M.B.Ch.B.

J. and A. Churchill.

"Chavasse's Advice to a Wife." Fifteenth edition. Revised by G. Drummond Robinson, M.D., B.S., F.R.C.P.

Sisleys, Ltd.

"The Over-production of Women." By Mrs. Erskine.

Henry Frowde and Hodder and Stoughton.

"Infant Feeding." By J. S. Fowler, M.D., F.R.C.P.

THE HOSPITAL April 3, 1909.

*Name* ..............................................................

*Address* ..............................................................

..............................................................

**This Coupon must accompany manuscript or contributions intended for The Hospital.**

